# Draft Genome Sequence of the Methanotrophic Gammaproteobacterium *Methyloglobulus morosus* DSM 22980 Strain KoM1

**DOI:** 10.1128/genomeA.01078-13

**Published:** 2013-12-19

**Authors:** Anja Poehlein, Jörg S. Deutzmann, Rolf Daniel, Diliana D. Simeonova

**Affiliations:** Genomic and Applied Microbiology and Göttingen Genomics Laboratory, Georg-August University Göttingen, Göttingen, Germanya; Civil & Environmental Engineering, Stanford University, Stanford, California, USAb; Laboratory of Microbial Ecology, University of Konstanz, Constance, Germanyc

## Abstract

Here, we report the draft genome sequence of the methanotrophic gammaproteobacterium *Methyloglobulus morosus* DSM 22980 strain KoM1, which is proposed to be the type species for the novel genus *Methyloglobulus*. The genome (4.143 Mb) consists of a single circular chromosome and harbors genes for 2-aminoethylphosphonate (ciliatine) biosynthesis.

## GENOME ANNOUNCEMENT

The methanotrophic gammaproteobacterium strain KoM1 was isolated from profundal sediment from Lake Constance by initial cultivation in opposing gradients of methane and air. KoM1 is an anaerobic, methane-oxidizing bacterium that belongs to the type I methanotrophs. However, strain KoM1 grows best at reduced oxygen tensions (partial O_2_ pressure [pO_2_] = 0.05 × 10^5^ to 0.1 × 10^5^ Pa), whereas higher tensions result in delayed or inhibited growth. Based on 16S rRNA gene sequence identity and similarity of the marker genes *pmo*A, *nifH*, and *mdh* from other methanotrophs, strain KoM1 is considered the type strain of the new genus *Methyloglobulus* and the new species *Methyloglobulus morosus*. Here we present the draft genome sequence of strain KoM1.

Methanotrophs play a crucial role in mitigating greenhouse gas emissions due to their activity as biofilters for methane at the oxic-anoxic interface of soils and sediments (1). Community structures of methanotrophs differ among environments, but the underlying physiological properties of the different strains determining these differences are largely unknown and need to be resolved to understand these important regulators of methane emissions. The genome sequence of strain KoM1 provides the first step to understand the metabolic capacities of this methanotroph and explore its environmental role.

Genomic DNA of *M. morosus* was isolated with the Puregene core kit B (Qiagen, Hilden, Germany). Extracted DNA was used to prepare shotgun libraries with Illumina sequencing as recommended by the Illumina platform manufacturer (Illumina, San Diego, CA). Sequencing resulted in 7,722,867 paired-end Illumina reads and coverage of 185.58-fold. The initial hybrid *de novo* assembly performed employing the MIRA 3.4 software resulted in 184 contigs. The genome of *M. morosus* strain KoM1consists of a single chromosome. The overall G+C content was 47.31 mol%. YACOP and GLIMMER (2) software tools were used for automatic gene prediction, and RNAmmer and tRNAscan were used for identification of rRNA and tRNA genes, respectively (3, 4). The functional annotation of the protein-encoding genes was carried out with the IMG/ER (Intergrated Microbial Genomes/Expert Review) system (5).

The total number of predicted genes was 3,946, of which 3,885 are protein encoding. Overall, 2,896 of the open reading frames (ORF) (73.39%) were assigned to functions. In addition, 37 tRNA genes were predicted. The genome contained a complete *pmoCAB* operon encoding particulate methane monooxygenase and two operons related to the *pmxABC* operon encoding an unknown methane monooxygenase-like protein (6), but no soluble methane monooxygenase genes were identified. As typical for a type I methanotroph, the genes encoding C_1_ assimilation via the ribulose monophosphate (RuMP) pathway were present. An intact oxidative tricarboxylic acid (TCA) cycle and an incomplete serine cycle were also found to be encoded in the genome. KoM1 is able to fix nitrogen, as confirmed by the presence of a putative *nif* gene cluster. Among 2,242 strains of *Gammaproteobacteria* with sequenced genomes in the IMG database (as of 9 September 2013), strain KoM1 is one of 13 which possess potential genes encoding a 2-aminoethylphosphonate (ciliatine) biosynthesis pathway (7, 8).

### Nucleotide sequence accession numbers.

This whole-genome shotgun project has been deposited at DDBJ/EMBL/GenBank under the accession number AYLO00000000. The version described in this paper is the first version, AYLO01000000.
